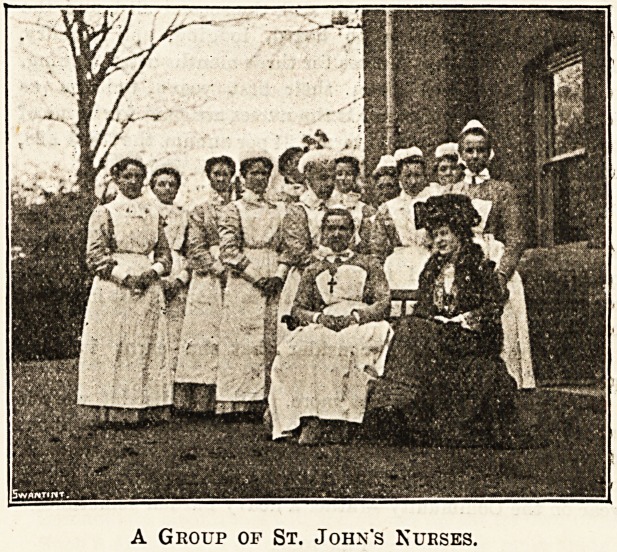# The Hospital. Nursing Section

**Published:** 1904-06-18

**Authors:** 


### The Hospital.

### Nursing Section.

**Contributions for this Section of "The Hospital" should be addressed to the Editor, "The Hospital" Nursing Section, 28 & 29 Southampton Street, Strand, London, W.C.**

No. 925.—Vol. XXXVI. *SATURDAY, JUNE* 18, 1904.

### Notes on News from the Nursing World.

### THE "ALEXANDRA" NURSES.

The annual meeting of the Soldiers' and Sailors' Families' Association was held on Thursday last week, Prince Louis of Battenberg in the chair. Queen Alexandra, the President of the Association, sent a gracious message wishing every success to the meeting, and on the motion of General Sir John Ardagh the report of the council was adopted. With regard to the nursing branch, we are glad to learn that the work continues to increase, and that the reports received from various stations contain expressions of satisfaction at the benefits derived from the services of the "Alexandra" nurses. In some cases where it was thought impracticable to establish such nurses, it is now stated that it would be impossible to do without them. During last year five additional nurses have been employed, namely, one in Plymouth, Deal, and Dublin, and two in Malta. Moreover, two commenced work in Edinburgh and Leith on January 1, 1904. Twenty-four nurses are now at work in the interests of the Association at a cost of about £2,200 per annum. The only unsatisfactory feature is that a nurse who was engaged for Salisbury Plain branch on January 1, 1903, was dismissed on the 8th of the following March, and up to now no successor has been appointed.

### QUEEN ALEXANDRA'S NURSING SERVICE.

It is satisfactory to learn on the authority of the Army Council that the standing orders of the Royal Army Medical Corps are shortly to be amended so as to make clear the position of matrons, sisters, and staff nurses of Queen Alexandra's Imperial Military Nursing Service. Meantime, it is notified that as regards medical and sanitary matters, the work in connection with the sick, the members of the service are to be regarded as having authority in and about their hospitals next after the officers of the Royal Army Medical Corps, and are at all times to be obeyed accordingly, and to receive the respect due to their position. Instructions given by them in connection with their duties are to be unhesitatingly complied with; and the Army Council request that this must be carefully impressed upon all non-commissioned officers and men of the Royal Army Medical Corps and others employed in military hospitals.

### THE NEW POOR-LAW NURSING ORDER.

In the House of Commons on Tuesday, Sir Harry Samuel asked the President of the Local Government Board whether boards of guardians of England and Wales would be given an opportunity of expressing their opinions upon the contemplated order to be issued by the Board consequent upon the recommendation of the Departmental Committee upon the nursing of the sick poor in workhouses; and whether a draft of such order could be furnished to the Poor-law Union Association of England and Wales before the same was made absolute. Mr. Long, in respect to the first point, stated that he had received and duly considered a large number of representatives from boards of guardians on the report of the Departmental Committee, and cannot therefore undertake to afford them any further opportunity of stating their views on the subject. But he promised to take into consideration the suggestion in the latter part of the question. We hope that he will comply with it, unless the concession means a good deal more delay. The time for the issue of the order has certainly arrived.

### NURSING IN INDIAN PROVINCIAL HOSPITALS.

According to a correspondent, who is herself a superintendent nurse in one of the best known provincial hospitals in India, the state of nursing in these institutions is at present deplorable. This is sure to be the case so long as the probationers are of an unsatisfactory character; and our correspondent, whose experience and position entitle her testimony to be regarded as reliable, intimates that the troublesome *protégée* who has failed in all other callings is the sort of person who enters as probationer in a provincial hospital. She suggests that the first move for reform should come from the trained nurses in India who hold posts similar to her own, and we think that she is right. A conference of superintendent nurses in the various presidencies could hardly fail to bring about some useful practical results, and we hope to learn that this has been arranged. There is no reason why the provincial hospitals in our Eastern Empire should not share to the full extent in the progressive movement, but the initial step in such a direction must be taken by those who are responsible for the nursing. In an appeal for funds issued this week by the Up-Country Nursing Association for Europeans in India, we notice it is stated that quite lately the managers have found it a difficult matter to comply with pressing requisitions for more nurses for the Punjab and the United Provinces. We recommend that the Association should be represented at any conference which may take place. Though their special business is to place nurses at certain central stations where their services will be readily available, the organisation is clearly concerned in any movement to improve the standard of nursing in India.

### COTTAGE NURSES AND MIDWIFERY.

At the annual conference of the Holt-Ockley System of Affiliated Benefit Nursing Associations for the supply of cottage nurses on the Holt-Ockley system last week, Dr. Boxall spoke of the Midwives Act and its general effect upon nursing associations and the general community. He approved the suggestion that every certificated midwife should have had some general hospital training. We go further, and agree with the Sister Superior of the Community of the Nursing Sisters of St. John the Divine, who, it will be seen from the report of an interview with our Commissioner, urges that none but persons with general hospital training should be allowed to practise as midwives. This would do away with the possibility of girls of 21 becoming qualified under the Act.

### KING'S COLLEGE HOSPITAL AND ROMAN CATHOLIC NURSES.

The authorities of King's College Hospital have removed the regulation under which attendance at the Church of England services in the chapel was required. The principal, writing to Mr. J. F. X. O'Brien, M.P., on the subject, states that "all the rules requiring attendance at chapel, or in any way interfering with the religious freedom of nurses and sisters, were removed at a meeting of the Committee, held on May 11th last." Mr. O'Brien had threatened to oppose the King's College Hospital Bill in the House of Commons if the regulations were not abandoned.

### HOSPITAL-TRAINED NURSES AT STIRLING ASYLUM.

The appointment of Miss E. Satchwell to the post of matron of Stirling District Asylum, Larbert, is a suitable occasion for recognising the efforts which have been made by Dr. George M. Robertson, the medical superintendent, to hospitalise the institution under his control, and raise the standard of mental nursing. There are now at the Stirling District Asylum five hospital-trained nurses in charge of as many divisions on day duty, three being on the female and two on the male side. Half of the staff on the male side are women; and there is a very large night staff of male and female nurses, supervised by a night superintendent, who is also a hospital-trained nurse. Since these innovations have taken effect up to the present there have been two matrons, one for the female and the other for the male division; but owing to the retirement of Miss Gambley, matron of the female department, at the end of 21 years' service, Dr. Robertson has been enabled to proceed a step further towards the accomplishment of his ideals by the appointment of one matron in full charge of the whole asylum. Miss Satchwell, who was only recently selected for the matronship of the male department, will, no doubt, justify her promotion to this important position.

### PROPOSED EXTENSION OF THE ANGLO-AMERICAN HOME AT ROME.

As an appeal has been issued to the public by the managing committee of the Anglo-American Nursing Home at Rome for donations to enable them to purchase, at a cost of about £500, a plot of land in order to enable them to build a pavilion which would contain rooms suitable for patients who cannot afford the ordinary charges of the home and also accommodation for an increased number of nurses, it is pertinent to repeat the question asked by Lady Frederick Bruce at the last general meeting. Lady Frederick wanted to know how the patients could be lodged cheaper in a new building than in the existing home, but the chairman did not explain. Moreover, if there has been a difficulty in keeping up the staff of nurses hitherto, will it not be accentuated by an augmentation of the number? We are informed that five individuals who were eligible for free beds lately declined to make application for admission. If the committee find people refusing to fill free beds, how can they expect to occupy more beds which are not free?

### DRAWING-ROOM MEETING FOR NURSES.

By invitation of the Earl and Countess of Portsmouth a drawing-room meeting for nurses will be held at 16 Mansfield Street, Portland Place, on Friday, July 1, at 3.30 P.M. Sir William Collins, M.D., has consented to give an address at 4 P.M., and music and refreshments will, no doubt, add to the enjoyment of the afternoon. Any nurse can obtain a card by applying to Miss Hilda Dillon, 47 Oakley Street, Chelsea, S.W., but no one will be admitted without a card.

### CROYDON GENERAL HOSPITAL.

On Friday last an interesting function was performed at Croydon General Hospital by the Chairman, Sir Frederick T. Edridge, who presented to Probationers Margaret Watson and Grace Tuck first and second prizes, respectively, gained by them as the result of competitive examinations in anatomy and human physiology, following courses of lectures which had been given to the nursing staff during the season 1903–4 by Dr. Herbert Male and Mr. Gervase Newby, F.R.C.S. Sir Frederick, in making the presentation, expressed his gratification that the reports of the examiners spoke in complimentary terms of the keen interest evinced in their studies by the fifteen probationers, of whom, in addition to the prize-winners, Nurses Catherine Staines and Helen Legg were specially commended. In the name of the nurses and on behalf of the Committee he thanked Dr. Male and Mr. Newby for their kind services, both as lecturers and examiners, which everyone had so much appreciated. Lady Edridge was present with the matron, Miss Bird, and took part in the proceedings.

### CHRONIC PATIENTS IN THE UNITED STATES.

Some interesting details are given by Dr. Arthur Hallam Ring of the Sanatorium, Arlington Heights, Massachusetts, of the difficulties experienced by the authorities of that institution in obtaining nurses qualified to take care of chronic patients. Dr. Ring states that hospital nurses were tried first, but "they were impatient and felt that there was too little nursing and too much housework." Graduates of the asylum schools were next employed, but they had "a tendency to treat patients without consideration and as machines." The third move was the engagement of untrained attendants, who were instructed as occasion demanded, but these "no sooner became useful than they were carried off by some admiring patient." Accordingly, it was decided to establish a small nursing school with a one-year course, but one year was found to be insufficient, and after much thought and experience a second was added. The first year embraces anatomy and physiology, chapters on special subjects and nervous system being omitted, recitations one hour weekly, nursing recitations one hour weekly, lectures and practical lessons on massage, lectures and practical lessons on hydratics, lectures on medical gymnastics, lectures and practical lessons on cooking for invalids, and reading aloud for three months. In the second year there are further recitations and lectures on some of these subjects and, in addition, lectures and demonstrations in electricity, lectures on psychology, psychiatry, and the ethics of nursing. Great pains are taken to choose young women coming from good homes and with a high school education, and they are taught the art of entertaining as well as caring for their patients. No attempt, Dr. Ring adds, has been made to create a field for the nurses who are trained at the sanatorium, but the demand for them is already greater than the supply.

### THE COMING OF AGE OF A NURSING SOCIETY.

The Cathedral Nursing Society of Newcastle has come of age. At the twenty-first annual meeting the chairman, in moving the adoption of the report, observed that the Society appeared to have been very well supported by a large number of influential subscribers, and the financial statement justifies this view. The total income in the last 12 months was £1,952, as against an expenditure of £1,841. As to the work itself, during the year 1,743 cases were visited, 17,069 visits were paid, and 171 night nurses were employed, a considerably greater number than in the previous year. Each succeeding period the calls upon the society increase, and its value—the skill of its nurses and the Catholicity of its benefactions—is gladly acknowledged by the working men on Tyneside.

### A NEW DEPARTURE IN INDIA.

Miss Cornelia Sorabji has been appointed legal adviser to the Court of Wards, Government of Bengal. Her position will enable her to communicate directly with Purdah women, whose business relations with their male agents or managers are confined to conversations through a curtain or screen. This opens the door to fraud, and Miss Sorabji, who is a B.C.L. of Oxford and LL.B. of Bombay, has long advocated the employment of female legal advisers on the analogy of the system of female medical assistance organised by Lady Dufferin. She has now the opportunity of putting her proposals to a practical test.

### NURSES IN THE LAW COURTS.

Last week Mrs. Jane Dyer, a nurse, sustained a claim of £20 for services rendered, in the County Court at Barry, against a bank manager, who was one of the executors of a patient she had attended. The action was brought by consent, the defendant merely desiring a judgment of the Court before paying the money out of the estate. An action of a very different kind was brought in the King's Bench Division on Monday by a nurse named Florence Dreher, described by her counsel as an experienced nurse, to recover damages for breach of promise of marriage. The defendant is a tailor and outfitter at Richmond, and the evidence reflected discredit on both parties. In the result judgment was found for the defendant with costs, by direction of the Judge.

### A STRUGGLE FOR A REVOLVER.

Two nurses who were in attendance on Mr. George Whittall, a Kidderminster carpet manufacturer, have had a dreadful experience. The patient had undergone two operations recently, and was under medical treatment. Early on Sunday morning he seized a revolver, but his nurses struggling with him, wrested the weapon out of his grasp. He succeeded, however, in breaking away from them, and rushing upstairs into another room, found a second revolver, with which he shot himself through the head. We do not know that the nurses were to blame for loaded revolvers being in two rooms in the house of a patient, but the terrible result in this case suggests the exercise of care in removing any instruments of danger from accessible places.

### GOOD PROGRESS AT HARROGATE.

It would not be creditable to such a prosperous health resort as Harrogate if the District Nursing Association were unable to pay its way. The report which was carried at the annual meeting, on the motion of the Mayor, shows that the support given to the organisation is very satisfactory. The year started with a balance of £162 on the right side, and the balance at the end of it was practically identical, which means that the whole of the current expenses were defrayed by the subscriptions and donations received in the twelve months. Such a result is all the more to be welcomed because the work of the nurses was exceptionally heavy, many of the cases being serious enough to necessitate two, and in some instances three, visits daily. In the circumstances, it was very wisely resolved to transfer £25 from the balance in hand on the revenue account to the endowment fund.

### SAVED BY THE PROCEEDS OF A JUMBLE SALE.

In the report of the Droylsden District Nursing Association, which was submitted at the annual meeting, it was stated that the financial position was saved by the proceeds of the jumble sale. We agree with the comment of the chairman that this is not satisfactory, especially as it is not possible to have a jumble sale every year. All special efforts on behalf of nursing organisations are naturally welcomed, but there is sometimes a danger that the prospect of having help from a concert, or a sale of work, may cause the relaxation of efforts to increase, or even maintain, the list of subscribers. The insistence by the chairman of the Droylsden Association upon the vital necessity of personal work, and the strengthening of the number of volunteer collectors who bring in the normal income, is therefore to be commended.

### SHORT ITEMS.

Miss Agnes Margaret Pagan has been provisionally appointed staff nurse in Queen Alexandra's Imperial Military Nursing Service.—The committee and staff of West Ham and East London Hospital will hold the third annual "At Home" on Saturday afternoon this week.

### The Nursing Outlook.

"From magnanimity, all fear above;
From nobler recompense, above applause,
Which owes to man's short outlook all its charm."

### THE DANGERS OF AN IRRESPONSIBLE CHARITY.

A case of peculiar interest was heard lately in the Pocklington County Court, and was referred to in our pages last week. The story was not an unusual one: a nurse sent by the Wilton Beacon Benefit Nursing Association to an operation case on a labourer's wife, was accused of badly burning the patient by the careless application of hot water bottles. The defendants were Lady Margaret Bickersteth and Mr. J. J. Bickersteth, the Hon. Sec. and Hon. Treasurer of the Association, and Mr. Bickersteth conducted his own case and won. But the interesting point which came out in evidence was the nature of the nurses supplied by this "Benefit" Association. We quote from the report in the *Yorkshire Herald*:—

"Mr. Bickersteth addressed the Court, and explained that the Association was started by Lady Margaret Bickersteth fourteen years ago, and there were two hundred on similar lines in different parts of the country. The object was not to supply nurses, but they had tried to take the old village Gamp, who alternated between the sick-room and the potato field, and turn her into something very much better.

"His Honour said it seemed very much like a friendly society, in which there was some almost nominal payment which entitled the members to the services of a doctor. He had never heard that the patient had any cause of action against the society which supplied the doctor.

"Mr. Bickersteth, continuing, said he was not sure that a member of a club had a right of action against his fellow-members for the default of a common servant. Nurse Quest, who had formerly been an Association nurse for three years, was sent under the impression that the plaintiff wanted nursing up ready for the journey to Hull."

The danger of these charitable associations under the present system lies in the fact that according to the law no one can be held responsible in cases of mishap through the employeés' ignorance. The lives and future well-being of the sick poor are in the hands of a well-meaning association who, under the plea that any nursing is better than none at all, provide a cheap and inefficient service which effectually deprives the suffering poor from securing aid from the trained and skilled nurse.

There is no pretence in the case before us that the nurse sent was fit to take charge of an abdominal section, but whereas Lady Margaret and Mr. Bickersteth may be credited with knowing the difference between a trained and an untrained nurse, we doubt if the members of the Benefit Association know, and we strongly deprecate the taking of their pence and then supplying them with an inferior article. It was alleged against the nurse that she not only allowed the feet to be burnt, but then dressed them with rancid oil, and did not at once tell the doctor. How is some poor villager's wife to know what is happening to her if she is left in charge of such untrained nurses as this? Again and again we have protested against the "village nurse," and urged the adoption of the Queen's nurses in every part of England, Ireland, and Scotland. The responsibility devolving upon these women in out-of-the-way cottages is enormous, and it is only through the Queen's nurses that they can be kept up to the mark and in touch with modern methods, can be inspected and helped, and cheered in their lonely work. We hope Lady Margaret Bickersteth, though she won her case, will now see that the due protection of those she desires to help necessitates stricter and more trained methods. It is poor charity to run a concern that is not perfect, and for our part all our sympathy is with the unfortunate Mrs. Hayhurst. We hope that the other 1,335 families who belong to this benefit society will see to it that they may not be caught in like case. The idea that anything is good enough for the poor is a dangerous one when it comes to a question of life and death, or of the crippling of a limb to those who have to labour for their living. Two doctors had the courage to give evidence and state that they considered a fully-trained nurse ought to have been employed, but the evidence shows that the surgeon in charge of the case declared that he did not want the services of a highly-trained nurse. Nevertheless, the result proved that the employment of an untrained person brought about disaster.

Cannot doctors make a firm stand against these "sick-room and potato-field" Gamps of to-day? Surely, if Dr. Jefferson and Dr. Fairweather followed up their evidence by addressing the next annual meeting of the Wilton Beacon Benefit Association something might be done.

We do not blame the nurse; but we do blame all associations which supply half-trained or untrained nurses to the poor. There is no necessity for such doubtful philanthropy—it is perfectly easy to get Queen's nurses now if a decent salary be paid. The responsibility of those who foist ineffectual help on the poor villager in time of illness is enormous: let them look to it that remorse may not dwell with them in the end.

### Lectures Upon the Nursing of Mental Diseases.

By Robert Jones, M.D.Lond., B.S., F.R.C.S.Eng., M.R.C.P.Lond., Resident Physician and Superintendent of the London County Asylum, Claybury.

### LECTURE XIII.

It is very important that the nurse should always report to the medical officer in charge any change in the bodily and mental conditions of the patient, as well as in the "environment." To do this it is necessary that she should observe and remember the different functions of the body and how these react upon each other, and upon the patient's surroundings. Any fresh mental symptoms, new delusions, increased excitement, the use of threatening language, or unusual depression, any attempts to evade supervision, plans to escape, or conversation relating thereto, the occurrence of fits, where they begin, and whether there was loss of consciousness, or paralysis; or if several fits in succession took place. Her observation would lead her to distinguish between epileptic fits—beginning with the "cry" or accompanied by a scream, foaming at the mouth, with loss of consciousness —and apoplectic fits, with more or less complete and permanent paralysis on one side of the face and body, or hysterical fits, without loss of consciousness, with sobbing or emotional tears and no paralysis, or whether the fit was a faint or a syncope through cardiac failure, or whether it was a rigor, or an attack of malaria, or a shivering fit indicating the onset of pneumonia, or one of the infectious fevers. Any accident, fall, or struggle should be at once reported, however slight, as fractured ribs or serious internal injury may occur without any complaint being made by the patient. Any change in the habits, the appearance of unusual drowsiness or increased sleeplessness, or helplessness in regard to controlling the excretions, unusual thirst, voracious appetite, or refusal of food, loss of flesh, or any change in the bodily weight —which in institutions is often taken weekly—any sickness, or pain, inability to walk, or a dragging of the limbs, and any change in the general appearance of the patient, all these symptoms need special attention called to them. It is usual to consider everything that goes into and everything that comes out of the body, as well as any noticeable changes in the body itself and in the environment, to be worthy of report. What has the patient taken? Is food and drink voluntarily, easily, regularly, and properly taken, or has the patient swallowed anything harmful, such as bits of bone, pieces of glass, stones, leaves, or grass? Has there been apparent discomfort, pain, or sickness after food is taken? What is the character of the matter ejected? Is there pus or blood in it, or is it changed or unchanged food? Is the medicine or are the extras that are ordered taken satisfactorily? Are the excretions regularly voided? Is the urine normal in quality and quantity, and do the bowels act daily and naturally? Is there a cough with expectoration of mucus, pus, or blood? As to the body itself, any general changes, emaciation or gain in weight must be recorded. Is the skin normal in colour and are there any eruptions, bruises, ulcers, abrasions or signs of injury? Finally comes the environment. Are all doors and windows securely fastened, and are all medicines, lotions, poisons, applications, such as bandages or dressings, securely put away? Are all keys, scissors, knives, step-ladders with cords attached, under proper supervision? Are all matches, pocket-knives, string, boot-laces, window-cords, hooks, or anything with which the most ingenious devices of even a sane person could contrive to use for personal harm, or danger to others, carefully put away?

The insane are liable to the same diseases as the sane, with possibly a preponderance of breakdown from nervous diseases, ordinary forms of which are met with in asylums as well as in the general hospitals. There is often great difficulty in diagnosing diseases in the insane, as there are frequently no subjective symptoms, *i.e.*, definite complaints of pain or discomfort may be absent, and the patient may not be able to assist by modifying the respiration when told during examination of the chest, or assist in other ways by replying to questions hence the great assistance derived from the nurse's own observation in regard to changes which may be noticed in the patient's conduct or appearance. We shall refer to diseases as falling under the group of organs affected, such as the nervous, respiratory, digestive, and other systems. In dealing with the nervous system, it is proposed for the information of nurses to deal generally and broadly with diseases of its various divisions, viz., the (*a*) brain, (*b*) spinal cord, and (*c*) the membranes—the *meninges*. The commoner diseases of the brain, for which special nursing is needed, are paralysis, usually affecting the whole of one side, chorea, or "St. Vitus' Dance," epilepsy, and hysteria.

Paralysis occurring on one side of the body is caused generally by some injury to the opposite side of the (*a*) brain. Such injury may be due to a tumour or to the blocking up of an artery through a clot carried into or forming in it, or the rupture of an artery with hæmorrhage into the brain, the blood collecting in the substance of the brain, which is torn and ploughed up and its functions impaired in consequence. This is generally described as an apoplectic fit or a "stroke." The symptoms come on suddenly, the patient being previously in perfect health. There is loss of consciousness amounting to complete and total insensibility —a condition called "coma"—with stertorous or snoring breathing, indicating the depth of the paralysis. This state may end fatally after a very great rise in the temperature, probably from 108° to 110°; but if partial improvement occurs, one or other side is found to be limp and paralysed, and the sensation in the affected side greatly impaired or absent. If the right side is paralysed, there is most often great or complete loss of the power of speech. The treatment for the nurse in such a case is to see that the patient is kept quietly in bed with the head slightly raised, and that the patient is turned on one side or the other to prevent the mucus from accumulating in the lungs. Great care must be used in administering liquid food—the only form which can be taken—for it is apt to gravitate into the lungs, owing to loss of power to swallow, and this induces "aspiration-pneumonia," food or broncho-pneumonia as it is sometimes called. The patient must be kept very clean so as to avoid bed-sores, and when well enough to get up the paralysed limbs, which are colder than the others, must be kept warm and free from pressure to prevent sores occurring. When paralysis occurs the arm is the first to go and the last to recover. Some mental weakness—*dementia*—may occur after apoplectic fits which are also apt to recur. As to tumours of the brain, these may occur in persons of all ages and are occasionally met with in asylums. The most common in young persons and childhood are those due to tubercle, the others are caused by cancer or may result from specific disease. The symptoms are headache, vomiting, convulsions, and a change in disposition and mental powers, the mind changing more especially if the tumour is in the front part of the brain; possibly also there may be some affection of vision. The nurse's duties in such cases are to keep the room quiet, cool, and darkened, particularly if there is sensitiveness to light. Headache is relieved by cold applications to the head or the use of an ice-bag. Leiter's tube is also used, being a coil of small lead tubing rolled into the shape of a cap, through which ice-cold water is kept circulating. Careful feeding with liquid food is to be observed and the bowels may require enemata. In regard to the convulsions it will be of much assistance if the nurse has observed and can report as to the position of the head and the direction of the eyeballs, also in what part of the body the convulsions commenced—whether in the face or arm—and whether they are limited to one side or the other.

Chorea or St. Vitus' dance is a disease characterised by irregular jerky movements of the limbs on one, sometimes on both sides of the body. This form of disease occurs not infrequently among asylum patients, more especially in young boys and girls, or women during pregnancy. There is often mental depression and dullness with the jerky movements or there may be the excitement of mania associated with them. Absence of emotional excitement and disturbance, together with quiet surroundings are necessary, also rest in bed, or on a mattress on the floor if the convulsions be severe, otherwise patients may be jerked out of bed during the attack and suffer harm. Great care must be taken in feeding them, and sleep is to be encouraged as the movements cease during sleep, and rest is essential to these cases. The patient may even have to be held by the nurse in very severe cases in order to check the involuntary jerks. It is very necessary to keep the patient clean. With regard to epilepsy it has already been described, and hysteria merges into mania by such insensible gradations in cases of insanity that no further reference here will be made to either of these diseases.

In the disease of the (*b*) spinal cord, if the whole cord is affected, *i.e.* the [afferent and the efferent tracts, viz., those that conduct impressions to the brain and from the brain, paralysis or loss of power affects both sides, a condition to which the term paraplegia (*para*, beyond, and *plegia*, *stroke or paralysis*) is used. There is loss of control over the bladder and the rectum and the habits become defective; there is also a great tendency for "bed-sores" to occur. The symptoms are: complete loss of power over the legs, and when the disease occurs high up in the spinal cord complete loss of power over both arms and legs. This form of paralysis is soon fatal, but patients may last a long time when the legs only are paralysed. These are very trying cases to nurse as they require constant supervision to prevent bed-sores and inflammation of the bladder through involuntary distention of the organ or from decomposition of the urine. When only one tract of the cord is affected, if it be the afferent, which conveys impulses from without to the brain, a condition termed *locomotor ataxia* may result, and there is marked want of control to direct the movements of the arms and legs. Patients thus affected cannot tell the condition of their limbs unless they can see them; there is loss of general co-ordinating power, and the sense of muscular position is also absent—what is called *stereognosis* or the feeling of solidity. In the dark or when the eyes are closed patients totter and tremble, and may fall. There is also loss of common sensation, the feeling of touch being impaired, and there may also be present what are called "lightning pains" either in the chest, abdomen, or the limbs. These pains are most distressing and cause patients to believe that they are annoyed by electricity or unknown agencies, and they may in consequence develop painful and dangerous delusions. Such cases are not uncommon in asylums, and are considered greatly to resemble general paralysis, but the duration of the illness is much more chronic. The treatment is to prevent bed-sores, to be careful in feeding, and to regard with attention the regularity of the excretions, more especially the functions of the bladder. Rubbing, or a mustard plaster will often soothe the chest or stomach pains, and gentle but progressive muscular exercise is greatly recommended. A reference to this will be made later. When the efferent tract is affected, the tract which is motor in function and carries nervous impressions from the brain to the muscles, there is marked paralysis and often much wasting. Such a condition may occur in children or adults and the effects of rubbing, massage and electricity are often most satisfactory as tending in the absence of normal nerve currents to keep up the nutrition of the muscles. This form of treatment is found to be very efficacious when the whole of a nerve or a group of nerves is paralysed, and a short reference to these methods will be made in a later lecture, as the mental nurse is supposed to be able to apply such treatment when so directed.

In disease of the (*c*) membranes of the brain—*meningitis*—the management by the nurse is similar to that given in the treatment of cerebral tumours. There is great sensitiveness to light in these cases and also much pain. Great care should be exercised to encourage sleep, as to feeding and to keeping the patients clean.

### How to Become a Midwife: The Rules of the new Act Explained.

By E. L. C. Appel, M.B., B.S., B.Sc.(Lond.).

### INTRODUCTORY.

The Midwives Act, 1902, was passed in order to secure the better training of midwives and to regulate their practice. It came into operation on April 1, 1903, and it enacts that—

1. *After March* 31, 1905, no woman shall be allowed to call herself a midwife unless she is certified under this Act.

2. *After March* 31, 1910, no woman shall be allowed to practise as a midwife unless she is certified under this Act.

The carrying out of the provisions of the Act is entrusted to the Central Midwives Board, and the supervision of certified midwives throughout England and Wales devolves upon the local supervising authorities, each local supervising authority being responsible for the supervision of all midwives practising within its area.

A woman who desires to become a certified midwife and to practise midwifery in England or Wales needs therefore to know—

1. How she may become certified under the Midwives Act.
2. The rules and regulations issued by the Central Midwives Board, which the certified midwife will be required to obey.
3. Her relation and duties, as a certified midwife, to the local supervising authority of the area in which she intends to practise as a midwife.

### I.—CERTIFICATION.

Contents.—How to Become Certified *before* March 31, 1905—*after* March 31, 1905—The Roll of Midwives.

There are three ways in which a woman can become certified under the Act, provided her application for certification is sent to the Central Midwives Board before March 31, 1905.

After March 31, 1905, there is only one way in which a woman can become certified under the Act.

*Certification Before March* 31, 1905.

The three ways in which it is possible to become certified before March 31, 1905, are:—

FIRST WAY.—*For women who at the passing of the Midwives Act had been for at least one year in bonâ fide practice as midwives, and who bear a good character.*

The Act was passed on July 31, 1902. Any woman, therefore, who was in *bonâ fide* practice as a midwife from July 31, 1901 to July 31, 1902, who bears a good character, and who wishes to become certified, should proceed as follows:—

1. Write for the Midwife Forms 8 and 9.[1]

*Form* 8 is:—I hereby claim to be certified under Section 2 of the Midwives Act, on the ground that I have been in *bonâ fide* practice as a midwife since .................. I enclose the necessary certificates and the fee of ten shillings.

Dated this ... day of ......... 19...
Name in full ..............................................
Single, married, or widow ..............................
Full postal address ......................................

Form 8 must be filled in by the midwife.

*Form* 9 is:—I certify that........................has, to my personal knowledge, been in *bonâ fide* practice as a midwife since ........................, and that she is trustworthy, sober, and of good moral character.

Dated this ... day of ......... 19...
Name ..........................................................
Address .......................................................
Calling or position .........................................
Signature of applicant ....................................

Form 9 must be filled in by a Justice of the Peace, minister of religion, registered medical practitioner, or other person acceptable to the Central Midwives Board, and the midwife must write her name after the words "Signature of applicant."

2. Send these two Forms, 8 and 9 (*i.e.*, the application and the certificate), together with the fee of ten shillings, to the Secretary of the Central Midwives Board, 6 Suffolk Street, Pall Mall, London, S.W.

3. In return you will receive from the Central Midwives Board a certificate (Form 11), which is as follows:—

CENTRAL MIDWIVES BOARD.
(2 Edw. 7. c. 17.)

*No.* ...... *Date* ...............

We hereby certify that ................. is entitled by law to practise as a midwife in accordance with the provisions of the Midwives Act, 1902, and subject to the rules and regulations laid down in pursuance thereof, by virtue of having been in *bonâ fide* practice as a Midwife for one year prior to July 31, 1902.

.......................... } Members of
.......................... } the Board.
................. Secretary.

This makes you a certified midwife.

SECOND WAY.—*For women who have obtained a certificate in midwifery from the Royal College of Physicians of Ireland, from the Obstetrical Society of London, from the Coombe Lying-in Hospital and Guinness's Dispensary, or from the Rotunda Hospital for the Relief of the Poor Lying-in Women of Dublin.*

If you hold a certificate in midwifery from any one of these bodies, you should

1. Write for a Midwife Form 6.[2]

*Form* 6 is:—I hereby claim to be certified under Section 2 of the Midwives Act on the ground that I hold a certificate in Midwifery from the.............................................. which certificate I enclose herewith, together with the fee of ten shillings.

Dated this ... day of ......... 19....
Name in full ..............................................
Single, married, or widow ..............................
Full postal address ......................................

Form 6 must be filled in by the midwife.

2. Get a certificate of identity.

This certificate must by signed by a Justice of the Peace, minister of religion, or registered medical practitioner, or by the secretary of an institution (approved by the Central Midwives Board) of which the applicant (*i.e.*, the midwife) is a member, or is or was an employee, and must state that the applicant is the person to whom the aforementioned certificate in midwifery was granted.

3. Send Form 6 (properly filled in and signed) together with your certificate of identity[3] and your certificate in midwifery,[4] and the fee of ten shillings, to the Secretary of the Central Midwives Board, 6 Suffolk Street, Pall Mall, London, S.W.

4. In return you will receive from the Central Midwives Board a certificate (*Form* 10) which is as follows:—

CENTRAL MIDWIVES BOARD.
(2 Edw. 7. c. 17.)

*No.* ...... *Date* ...............

We hereby certify that...............................is entitled by law to practise as a midwife in accordance with the provisions of the Midwives Act, 1902, and subject to the rules and regulations laid down in pursuance thereof, by virtue of holding a certificate in midwifery from

(*a*) The Royal College of Physicians of Ireland, or
(*b*) The Obstetrical Society of London, or
(*c*) The Coombe Lying-in Hospital and Guinness's Dispensary, or
(*d*) The Rotunda Hospital for the Relief of the Poor Lying-in Women of Dublin.

.......................... } Members of
.......................... } the Board.
................. Secretary.

This makes you a certified midwife.

[1] All Midwife Forms (price 1d. each, postage extra) can be obtained from The Scientific Press, Ltd., 28 and 29 Southampton Street, Strand, London, W.C.

[2] See Note [1].

[3] *Certificate of Identity.*—The Secretary of the Central Midwives Board will, by comparison of the handwriting, or by such inquiry as he may think necessary, satisfy himself as far as possible of the applicant's identity.

[4] *Certificate in Midwifery.*—In the event of the original certificate in midwifery having been lost, a voucher from the accredited secretary or other agent of the certifying body must be sent, stating that the certificate was granted to the applicant on such and such a date.

(*To be continued.*)

### TRAVEL NOTES AND QUERIES.

BY OUR TRAVEL CORRESPONDENT.

THREE WEEKS' HOLIDAY IN AUGUST (Matt).—Judging from your address, you must, indeed, need a cheerful rest. I can tell you of two ladies, one in Brighton and one near to Glastonbury, who take paying guests at very moderate prices; both homes are cheerful, with young people. Your means are ample for either place. If you could find a friend to go with you, I think I could make a good arrangement for you, on very low terms, in Bruges. It would be a much greater change for you if you could go abroad, and you would store up pleasant remembrances of great variety to cheer you in the winter months. The three addresses I allude to are all private, so you must send me a stamped and addressed envelope, and I will send them by post. I do not arrange parties, and those organised by the usual agents would not suit you, because they are almost always for short periods.

### The Nurses of the Community of St. John the Divine.

AN INTERVIEW WITH THE SISTER SUPERIOR: BY OUR COMMISSIONER.

That admirably-equipped hospital at Morden Hill, Lewisham, is an outward and visible sign of the earnestness and enterprise with which the Community of the Nursing Sisters of St. John the Divine quietly pursue their devoted labours. The work of the Community, which is the training and employment of sisters and nurses for hospitals, private families, and the poor, is carried on partly by public subscription and partly by private effort. The Bishop of Rochester is visitor, and the patrons and honorary officials are all well-known personages, while the hospital is under the management of an influential committee. But it was not to ask questions about the nursing at St. John's Hospital so much as to obtain information respecting the training of the nurses generally by the Community that I requested Miss Beaver, better known as the Sister Superior of the organisation, to see me at the head-quarters in Drayton Gardens, South Kensington.

At the outset of our conversation the Sister Superior mentioned some interesting facts in connection with the origin of the movement.

### Miss Nightingale's Colleagues.

"The work," she explained, "was really started in different homes before the Crimean war. In 1848 at 36 Fitzroy Square; in 1852 at Queen Square, Westminster; and in 1860 at Norfolk Street, Strand. The principal promoters of the movement were Dr. Todd and Mr., afterwards Sir Bartle Frere. Among the subscribers from the outset were the father and brother of Miss Louisa Twining. The idea of the founders was to associate together a number of ladies anxious to pull up the nursing and make it better. The movement, of course, received a great impetus, when Miss Florence Nightingale went to the Crimea, taking with her 20 of St. John's nurses. Some of these died in the performance of their duties, but others took their places, remaining out there until the war terminated."

"After they came home," continued the Sister Superior, "the authorities of King's College Hospital applied to the Community to take charge of the nursing. That was in 1856, and King's College was the first hospital to open its doors to us; but we also supplied nurses to the Westminster Hospital during a cholera epidemic in the fifties, and from 1866 to 1883 the nursing at Charing Cross Hospital was done under our auspices. We also were responsible for the nursing at the Galignani English Hospital in Paris, founded by the Messrs. Galignani, and other works of the kind."

### Branches of Work.

"When did the sisters cease the nursing at King's College Hospital?"

"In 1883. It was in that year also that we separated from Norfolk Street, and took the title of St. John the Divine in order to distinguish us from the other community. We now, in addition to the private nursing, have, as you know, our own hospital at Lewisham with 41 beds, and the District Homes at Deptford and Poplar for maternity and general work. Last year the number of patients who were treated at the Deptford Home was 2,648, of patients attended in their own homes 572, and of midwifery cases 441. We enjoy the receipt of grants from both King Edward's Fund and Hospital Sunday Fund, which are both greatly needed and much appreciated."

### Training of Nurses.

"How many nurses are in training at the present time?"

"The number varies from fifteen to twenty. We do not care to exceed twenty. Nurses joining the Community enter the Lewisham Hospital after a month's trial, and must sign for three years."

"Do you get many applications in excess of your requirements?"

"I should say that out of every dozen applications two are suitable. With regard to age, I consider that 23 or 24 is the best for entering. There is one point which I should like to emphasise in connection with training. For many years now we have been doing maternity training, and I invariably decline to train any persons for maternity work who have not had three years' hospital training."

### Midwives of Twenty-one.

"You do not approve of young girls of twenty-one acting as midwives?"

"On the contrary, it seems to me that it is of vital importance that the good general nurse only, who has had her full training, should take up midwifery or maternity nursing. A woman who has done general nursing is more likely to care for the baby and for the mother than the girl who has merely had three months' maternity training. Indeed, I consider it essential that a nurse should know how to nurse general cases before she attempts to nurse a new-

A Group of St. John's Nurses.

born baby and the mother. Therefore, I not only do not allow any of our nurses to enter for maternity training until they have had general training for three years, but even then, we select the very best. In my opinion it is not possible to exaggerate the importance of having maternity nurses of the best available class. The sisters supported and nursed an important maternity hospital of twelve beds in Chelsea for nearly twenty years."

### Special Features.

"In what respect does the training by the sisters differ from ordinary training in a general hospital?"

"The training is not confined to the work in the wards. Our probationers enter on condition that they do what they are told, and after they are trained they are given private or district work as the case may be. Some do better in the wards, and others are much more adapted for outside work. They are almost all, if suitable, trained as district nurses. As there are no medical students at the hospital, they have many opportunities of becoming proficient in surgical nursing. There is a modern operating-room, and the sisterin-charge is always accompanied by some of the nurses when an operation takes place."

"When the three years have expired, do you desire or expect a nurse to remain with you?"

"I certainly desire it, because we employ no nurses who have not been trained by us, but it is not compulsory. The usual custom, at the end of three years, is for the fully-trained probationer to sign for a fresh term as a nurse of the Community.

THE CONVENTIONAL CERTIFICATE.

"They are not required to take any vows?"

"No, but they cannot break their engagements with the Community. We do not give certificates, but if a probationer who has finished her course does not stay on with us, or a nurse wishes to leave at the end of her two years of service, I am always willing to give letters of recommendation. I do not attach much value to the conventional printed certificate which means so little."

"Do you sometimes have nurses leave the Community and return to it?"

"No, except they leave from illness or family reasons. My oldest has just retired after 38 years of uninterrupted service with the Community. I do not like my nurses to leave, because I do not train them for other people but for our own work."

"Are the nurses paid from the commencement?"

"The first year they receive £10, the second £18, and the third £20, in addition to board, lodging, and laundry. They wear their own dresses for three months after signing, and then they are given their first year's uniform as assistant nurse. Afterwards the nurses are paid for terms of two years; for the second term £24 per annum, the third £28, the fourth £30, the fifth £32, and the sixth £35."

"I presume that £35 is the maximum?"

"Yes, and at the end of eleven years there is no more signing. They just stay on. As we are discussing the question of money, I may say that the cost to the Community of training a nurse for three years is not far short of £300."

"How much do you consider that she earns in that period?"

"Possibly £100, but not more. All the district work is money expended, and the upkeep of the hospital is about £2,600 a year. A few of the patients pay a small amount, but, practically, the hospital imposes a charge of £1,000 a year on the Community—rather a heavy burden sometimes."

PENSIONS.

"In the event of the nurses being unable to work after many years of service, is any provision made for them?"

"At the expiration of twelve years of service the sisters subscribe to secure them a pension of £10 per annum in the Royal National Pension Fund. We have been affiliated to the Fund from the beginning; and we consider it to be the very best possible scheme for helping nurses to help themselves and to provide for their old age. All our resident nurses also contribute for themselves, and are urged to do so by the sisters. If all nurses only realised how admirable the Fund is, and what liberal terms are offered, they would, I am sure, hasten to avail themselves of it. Our nurses are not fine ladies. Most of them belong to the middle class, and they turn out very good nurses."

THE RELIGIOUS QUESTION.

"Then there is the question of religion."

"I make the most searching inquiries as to the character of applicants, and ascertain that they are what they profess to be. They must be members of the Church of England."

"You mean communicants?"

"Yes, confirmed and communicants. There are plenty of other institutions for other people. But the only condition is that they are churchwomen. We ask heads of families engaging our private nurses to try and arrange for them to attend church."

INFECTIOUS CASES.

"Where do the private nurses live between their cases?"

"At Drayton Gardens. The nurses engaged in night cases sleep here, and, except in bad weather, it is an advantage for them. They could not sleep in the flats. There is a nurses' home at Lewisham for the hospital staff."

"Do the private nurses attend all kinds of cases?"

"Yes, including infectious cases. There is no difficulty about disinfection. The nurse usually does the disinfecting in the house, and she disinfects herself too. I subscribe to the Fever Hospital in Liverpool Road, and if one of our nurses contracts fever, she is sent there. The most liberal arrangements are made, the treatment is admirable, and the utmost kindness is always shown.

THE SISTERS.

"How many nurses at present belong to the Community?"

"There are about 60 nurses, but with sisters and associates the number exceeds 80. The sisters are not only all trained nurses, but have all been trained at St. John's. They enter as lady pupils, or paying probationers."

"Necessarily for three years?"

"No one can be a sister who has not been trained for three years, but we have a few lady pupils who come for a shorter time. The object of some is just to get a little training to enable them to help in their parishes or homes. I prefer to train nurses, but I am glad to have three or four lady pupils. They pay 15 guineas a quarter, and it all helps. I advise the same age, but if ladies like to come for a little training earlier or later, I do not mind."

"Where are the sisters stationed?"

"I like to have two at the mother's home with myself, though sometimes I have to spare them to supplement other portions of the work. At the hospital there is a sister in charge, a house sister, a night sister, and four ward sisters. The district and maternity homes require two midwife sisters in each, occasionally three; and two or three general staff nurses. One sister, with a nurse, is always at the convalescent home in Littlehampton. I am giving you the numbers actually required under any conditions, but to keep everything going, and to provide for rest and change of air, we want more than these. Apart from the private staff there are about a dozen trained nurses in full work at the different places. My aim is that the staff should be taught to fill any vacancy that may occur, so that the whole machinery may run smoothly, and undue pressure anywhere be avoided."

REGISTRATION AND CHARACTER.

"Do you care to say anything about registration?"

"I consider it essential that a midwife or maternity nurse should have a certificate, but that certificate only means that she is qualified to do her midwifery or maternity work. So far as the trained nurse is concerned, I have thought a great deal about registration, but I cannot see how it can get hold of the character of a woman, which, after all, is the most vital matter. A nurse's life cannot be followed up after she has been registered, and the higher the standard of the hospital where she is trained the less a bad woman need try to keep it up. I am very anxious that something should be done to eliminate the black sheep, and if more independent gentlewomen could be got to help the nurses who require to be propped up it would be better. But the nurses who can stand by themselves do not want registration. If any of the sisters of our Community were asked if they would like it, they would be very much surprised."

### Preparation for an Operation for Appendicitis.

### EXAMINATION QUESTIONS FOR NURSES.

EXAMINATION QUESTIONS FOR NURSES.

THE question was as follows:—If called upon suddenly—say, with 12 hours' notice—to prepare for an operation for appendicitis in a private house where means are limited, how would you proceed both with regard to the patient and the available rooms?

FIRST PRIZE.

The best plan when going to a private house to prepare for an operation, especially when time is limited, is to write down all the things required; this can be done in the cab or train. Also write a separate list to give the people in the house of the things they can get ready for you. On arrival, first see to the sterilising of the water, as plenty is always required. See that the pans or kettles are well cleaned, also the jugs in which to receive the water when boiled. Then turn your attention to the patient. Give her a good injection of soap and water, to be followed two hours later by a wash-out of sterilised water. Then prepare the site of the operation. Remove all hair, scrub well with soap and water, rub over with turpentine, scrub again; finally, apply a compress of lint wrung out of 1–40 carbolic lotion, cover with protective, and secure with a binder.

Now see to the room in which the operation is to take place. Select one with a bay window, if possible, as this gives better light; paint the lower panes with whiting to prevent anyone seeing in. Remove all furniture not required, curtains and tapestry to be taken down.

A large dust sheet spread over the carpet is a good plan, instead of taking up the carpet, thereby causing more dust. Dust the window-sills, doors, and skirting-boards with a cloth wrung out of 1–20 carbolic.

Have the room warm, but not too hot—65°–70° Fahr.

Your table must be strong and steady, about 30 inches in height, 6 feet in length, 20 inches breadth.

This must be well scrubbed and covered with an old blanket and mackintosh folded in the centre, allowing the ends to fall over the sides of the table into buckets placed there to catch any fluid.

Have footstools or large books, in case the surgeon wants the table raised.

Two smaller tables will be required for the instruments, these must be placed within reach of the surgeon on his right hand.

Have plenty of nail-brushes and soap.

At the other side of the operating-table, out of the way, have another table with three basins for the sponges or swabs.

Plenty of towels will be wanted; these sterilise by placing in a steamer or fish-kettle and allowing the water under them to boil 20 minutes.

Sterilise all basins, etc., to be used by washing them in 1–20 carbolic.

Record on a piece of paper on the wall the number of sponges to be used.

Have ready bandages, expectoration cup, brandy, strychnine and hypodermic syringe.

Give the patient a drink of beef-tea four hours previous to the operation, then get her dressed in a warm night-gown, bed-jacket and stockings.

See that the bladder is emptied just before the surgeon comes.

"INDEFATIGABLE."

SECOND PRIZE.

I should remove all unnecessary furniture, ornaments, curtains, and bed-hangings. Twelve hours' notice would not be long enough to allow of taking up carpet and letting the dust settle in time; so should wipe it over with a new house-flannel wrung out in a pail of water to which had been added some disinfectant as Jeye's sanitas, or carbolic. Then cover over carpet with old sheets secured by drawing-pins; walls, ledges, furniture, and bedstead wiped over with a duster wrung out of sanitas and water. Bed made up with mackintosh and drawsheet, pillow for head, a bolster rolled in a sheet put across centre of bed and tightly tucked in on either side of the mattress to support patient's knees. An old-fashioned bonnet-box cut in half would act as temporary cradle to keep weight of clothes off abdomen if unable to procure a proper one; one or two hot-water bottles well covered with flannel. Have a fire burning with kettle on the hob. Temperature of room 70°. A kitchen- or two dressing-tables would substitute an operating-table covered with a folded blanket and sheet, pillow for head, and a light blanket to cover over patient. Two bricks, books, or boxes at hand, if wanted, to raise end of operating-table.

One table with pie-dishes or soup-plates for instruments, another for sponges, with two large basins (all dishes and basins well scalded out), one small table for anæsthetist with a soap-dish, towel and smelling-salts.

Foot-bath, or pail under the table, as receiver for fluid or soiled dressings. A good supply of hot and cold sterilised water. A dozen old towels, four basins, steriliser (a new fish kettle would do). Three sterilised towels. A nutrient of beef-tea, some brandy, bottle of saline solution, 1 drachm to the pint of boiled water.

Would give patient warm bath, including the head; if too ill to allow of this, would thoroughly wash patient over in bed between blankets. After shaving the pubes, should well wash the abdomen with soap and water, paying great attention to the cleanliness of the umbilicus. Sponge over with turpentine, and again with perchloride 1-2000, putting on a compress wrung out of perchloride 1-2000. Cover with jaconet, and keep in place with a many-tail bandage.

Should change compress again two hours before operation. Unless otherwise ordered, should give cup of beef-tea four hours before operation.

If patient has false teeth remove them. Hair done in two plaits.

Have ready night-dress cut up the back, flannel drawers, bed-jacket, bed-socks. See that the bowels are well emptied by simple enema. If unable to pass urine, it should be drawn off by catheter.

"CROMWELL."

THE PRIZE WINNERS.

The standard of excellence attained this month is in advance of previous competitions. A large number of papers have been sent in, and the average is decidedly good. The two selected for prizes show practical foresight, especially that by "Indefatigable." She is evidently a nurse who wastes no precious time, that spent in cab and train is to be successfully utilised. She mentions rather a low height for an operating-table, but means to have provision for increasing it if necessary. Both she and "Cromwell," the winner of the second prize, wisely foresee the dust occasioned by removing a carpet, and propose to cover it instead with sheets. Too much energy for immaculate cleanliness will sometimes defeat its own object. Such a disturbance of old-established dirt as must abide in an aged carpet is not to be thought of with only a few hours' grace before you. A great many nurses see this evidently, but only speak of pinning down sheets under the operating-table with a view to protecting the carpet, but it is necessary to have the entire fabric covered, so that the friction of the attendants' feet may not send up dust into the atmosphere.

HONOURABLE MENTION.

This is gained by "Bunnyens," a nurse who seems to have chosen a strange name but who sends an excellent paper, "Quetta," and "Vigilante."

WRONG ANSWERS SENT IN.

Will nurses please to note that questions headed "Questions for Nurses in the Colonies and Abroad Generally" are not to be answered by nurses in the United Kingdom.

QUESTION FOR JUNE.

Give an account of what you would consider the most satisfactory room in which to nurse a patient with a protracted illness, and how—speaking broadly—you would like it to be furnished. Ordinary maladies, not those of an infectious nature, being under consideration.

THE EXAMINER.

### Everybody's Opinion.

[Correspondence on all subjects is invited, but we cannot in any way be responsible for the opinions expressed by our correspondents. No communication can be entertained if the name and address of the correspondent are not given as a guarantee of good faith, but not necessarily for publication. All correspondents should write on one side of the paper only.]

### STATE REGISTRATION OF NURSES IN SOUTH AFRICA.

"A South African Matron" writes: For some years we have had State Registration for trained nurses and midwives in South Africa. So far it has made very little appreciable difference in the nurse's status. We still have many Gamps with us, and half-trained nurses; at the same time if the public wish to be sure of the quality of nurse employed they can always find a list of her certificates in the "Medical and Pharmacy Register for Colony of the Cape of Good Hope." The benefit to the nurse is that she can sue for her fees in a court of law. It also gives nursing the status of a recognised profession. A nurse is put upon the Register after producing a certificate of two years' training in a general hospital of over 20 beds (the towns in South Africa are small), and passing the Council's own examination, which is fairly stiff, written and *vivâ voce*, conducted by two qualified medical men. The Council could of course remove the nurse's name from their list for misconduct.

### THE ROYAL NATIONAL PENSION FUND FOR NURSES.

"Ex-Policy 3523" writes: If you can spare a little space in your valuable paper, I should like to express in a few words my great appreciation of the Pension Fund for Nurses. I joined it ten years ago, and now, when I am obliged to retire from active work owing to ill-health, I have a little nest-egg of £121 to call my own. Of this, £14 14s. has been added by compound interest and bonus. I can honestly say that had it not been for the quarterly premium which had to be paid, most likely I should have saved nothing during those years; but I should have spent all my earnings on dress, amusements, and gifts to relatives, which were thought lightly of, when it was known the fees were good, and work plentiful. For the sake of other nurses I write my experience; the younger a nurse is the lower the premiums, and the more she will save; then, when the rainy day comes, or possibly marriage, she will have something to call her own. It is also a savings bank, for after two years she can withdraw her money if she wishes, or she can will it to whom she likes in case of death. I believe the lowest pension is £6—the premiums of which are small enough to meet the salary of the youngest nurse, and the advantages are just the same. The Secretary is most kind and courteous, and always ready to explain any difficulty and answer any questions or to give valuable advice. I hope that others may profit by my experience, and reap the same benefit—hence my apology for this letter.

### THE THREE GRADES IN WILTSHIRE.

"Inquirer" writes: I am not a nurse, but a general reader of your paper, and take an interest in the work, and might I ask how the Wiltshire Association intend to assist the three grades of nurses mentioned at the meeting, in Salisbury, in your issue of June 4. From the note in The Hospital this is not plainly set forth. If it is to improve the salaries, especially of the highly-trained nurse, the intention is most laudatory, and will meet with universal support, as these nurses are much underpaid. Whilst the highly-trained nurse may be needed for the towns, every district now requires her equally, for medical men in the country districts are few and far between. I am acquainted with a large district, near Salisbury, where a nurse who has been fully trained in maternity, fever, mental and surgical work gets £70, and a cottage. Now, after paying a servant and keeping her, replacing broken articles in the house, providing her own uniform, etc, what can be left either for taking a holiday, subscribing to the Pension Fund, or otherwise providing for a rainy day, that is to say, if she provides herself and the servant with substantial food, which is absolutely essential when both night and day work has to be performed? The Wiltshire Association will do a good work if they bring this matter home to the ladies of the county, and impress upon them the fact that at present the highly-trained nurse is not adequately paid for her professional services. In fact, the ordinary domestic servant has more money in hand, at the end of the year, than the woman who has spent many years in training. This is, in my opinion, radically wrong and requires re-adjustment.

### NURSING IN PROVINCIAL HOSPITALS IN INDIA.

"A Superintendent Nurse" writes: Would you kindly allow me through your paper to draw attention to the present deplorable state of nursing in the greater number of provincial hospitals in India, in the hope that some reader at present in India, or who has been there, may be able to suggest some practical remedy. If we omit the large hospitals in Bombay, Calcutta, and Madras, why is it that in the few provincial hospitals where the training of probationers has been attempted it has not been a success? I do not think that any one who knows about the matter will disagree with me when I say that, with rare exceptions, the nurses are not what they ought to be from a nursing point of view. Why is it that we cannot get a better class of girls to take up nursing? Speaking lately to the superintendent nurse of a hospital where probationers are taken, and where they find it very difficult to get them to stay to complete their training, I asked why it was that there was this difficulty? Her reply was to the effect that they (I suppose she meant the committee) find it difficult to get probationers and they take anyone who offers. These stay for a little time, then they get tired and leave. Another medical worker connected with a large general hospital, in reply to my question as to what qualifications were required in girls entering for training, said: "The staff nurses must be pure European, though trained in India, but anyone will be taken as a probationer except pure Indians." This being the case, one need hardly wonder that so many people out here think so little of nurses, and if any lady has a troublesome protegé who has failed in other professions, there is all the more reason to think that she will do for a nurse. Something ought to be done to alter this chaotic state of things. And I believe if anything is to be done the first move must be made by the trained nurses in India holding posts as superintendent nurses. Would it be possible to have a conference of these nurses at some centre and discuss the matter? True, distances are great, which makes travelling expensive; but this difficulty could be overcome to a great extent by having two or three centres in different parts of India. I hope that some of your readers will help with suggestions.

### TWO MEDICAL MISSIONS IN BERMONDSEY.

Dr. Lena Fox, of Bermondsey Medical Mission, 44 Grange Road, Bermondsey, S.E., writes: My attention has just been called to a paragraph in your issue of May 28th, in which it is stated that the work I am carrying on in connection with the above medical mission has taken the place of the Church Missionary Medical Training Home in Bermondsey. This is far from the case, and I am glad to say that the Church Missionary Society Training Home is in full operation, although it was feared at one time that for financial reasons the work would have to be largely curtailed. I have seen so much of the poverty and suffering amongst the women and children of this greatly congested district of South London, that I felt called upon to live amongst the people and to endeavour to the utmost of my power to alleviate their sufferings, and there is ample scope for many workers. The Church Missionary Society are carrying on an excellent and much-needed training home, and anything I can do to assist them will always be at their command. We, however, require assistance both as regards financial help and in an increased number of lady workers and students. A comfortable home is provided for them, and training given in medical, surgical, maternity and district work, together with lectures in dispensing combined with practical work. Any help will be most gratefully acknowledged.

[We regret that the fact of Dr. Lena Fox leaving the

Medical Mission of the Church Missionary Society and starting one of her own, conveyed the erroneous impression that the latter had taken the place of the former.—ED THE HOSPITAL.]

A RELIGIOUS QUESTION AT NICE.

THE REV. JOHN FITZPATRICK writes from 11 Rue du Congrès, Nice: I beg the hospitality of your columns to vindicate, in as few words as possible, the cause of three nurses who have just been turned out of the Nursing Institute at Nice. It is admitted that our nurses have a certain right to be re-engaged, if they wish it, unless there is some fault to be found with their personal or professional conduct. One of these nurses has been here for four or five seasons, a second has been here for two, while the third has spent but one winter with us. No charge has been made or can be made against any one of the three; and yet they have all been categorically refused a re-engagement. These nurses are all Catholics, and all converts to the Catholic Church; and, as many nurses are Catholics born, and many, against their worldly interests, following the dictates of conscience and the right of private judgment, join the Catholic Church, this matter concerns much a large number of your readers. These, and the public generally, as it seems to me, ought to know the strange thing that has happened in our midst. As the English-speaking priest here I have had, and have, a very serious quarrel with the management of our nursing institute, because of the hindrances that were put in my way when, in the discharge of my priestly duty, I visited the home. But none of these nurses has at any time had anything to do with these Catholic in-patients, or has taken any part in my quarrel. Yet, when it began, one of the nurses was told that no more Catholic nurses would be taken, if there were to be difficulties with me. Some time afterwards, when another nurse told the superintendent that she intended to be received into the Catholic Church, she was informed in her turn that if she did she would not be employed again by the institute. Both these things are notorious, and the threats were recorded at the time, though it was supposed that they were idle words and were spoken in ill-temper, for the rules of the nursing institute make no conditions about religion, and it is fully admitted, at least in words, that it has nothing whatever to do with such matters. No doubt, it is this nurse who is the greatest criminal; for, seeking first what her conscience told her was the Kingdom of God, and being not more solicitous for the morrow as to what she should eat than are the birds of the air of which our Saviour spoke, or more solicitous as to her raiment than are the lilies of the field, she has carried out her intention and has been received here at Nice into the Catholic Church. Of the two other nurses who have been so rudely pushed into the same boat with her, one became a Catholic in Paris seven years ago, and the other in London about as many months ago. Now, the problem is: why should the one be made suffer for her change of faith; and if she is to suffer, why should the two others be punished also? Or, again, why should any one of the three be made to pay for my real or supposed delinquencies, with which they have not had anything whatever to do? I ask your help, in the name of common justice, and in the name of English fair play which, it seems to me, ought to be practised even on the Continent, to expose this wrong-doing as it richly deserves.

### Presentations.

ROYAL NATIONAL HOSPITAL FOR CONSUMPTION FOR IRELAND.—Miss Hoadley, the late lady superintendent of the Royal National Hospital for Consumption for Ireland, was, on the eve of her departure for Coventry, the recipient of a handsome silver tea service and tray from the staff and patients of the hospital and her personal friends. Dr. Steede, who made the presentation, referred to the loss which both the hospital and her friends would sustain by her departure, and expressed on behalf of all the hope that she would in her new sphere earn even more popularity and esteem than she gained for herself during her period of administration at Newcastle.

### Appointments.

BEDFORD COUNTY HOSPITAL.—Miss Helen M. Mattocks has been appointed night sister. She was trained at the Royal Albert Hospital, Devonport.

DUMFRIES AND GALLOWAY ROYAL INFIRMARY.—Miss Eleanor Steenson has been appointed surgical sister, and Miss Lizzie Strath night sister.

HUNTINGDON COUNTY HOSPITAL.—Miss E. M. Crawford has been appointed matron. She was trained at Westminster Hospital, where she has since been staff nurse, sister-in-charge, and assistant matron. She has also been night superintendent (*locum tenens*) at Staffordshire General Hospital, Wolverhampton; matron (*locum tenens*), at the General Hospital, Dover; and matron of the Hospital at Hamilton, Bermuda.

ST. MARY (ISLINGTON) INFIRMARY, HIGHGATE HILL.—Miss Laura Lindsay has been appointed staff nurse. She was trained at St. Mary (Islington) Infirmary.

TAMWORTH ISOLATION HOSPITAL.—Miss Sarah Trainer has been appointed matron. She was trained at Ashton-under-Lyne District Infirmary, and has since been nurse at the Sanatorium, Marsden Road, Burnley.

WARRINGTON UNION.—Miss Mary Fanny Caunt has been appointed charge nurse. She was trained at Nottingham Union Infirmary.

WHARFEDALE UNION JOINT ISOLATION HOSPITAL.—Miss Minnie Dunbar has been appointed charge nurse. She was trained at the Royal Hospital, Belfast, where she was afterwards charge nurse. She has also been charge nurse at Brook Hospital, Shooter's Hill, charge nurse at Govan Parochial Hospital, Glasgow, and charge nurse at Mirfield Fever Hospital, Yorkshire.

### Novelties for Nurses.

BY OUR SHOPPING CORRESPONDENT.

### A NURSE'S CATALOGUE.

I HAVE received a copy of Messrs. Gayler and Pope's catalogue. It most fully illustrates the uniforms and nursing requisites which are to be found at their establishment, High Street, Marylebone. The uniforms portrayed are of neat and serviceable design, and the prices are remarkably low. Shoes, aprons, collars, cuffs, ties, dressing-gowns and jackets, and non-uniform costumes are included in the list. It is a convenience to nurses to find that all the simple requisites of the sick-room, from instruments to furniture, can be inspected when other purchases are made, and the catalogue further comprehends maternity outfits, layettes and travelling trunks and requisites. The establishment is conveniently situated not far from the Bond Street Tube Station, but those who cannot pay a personal visit, will do well to send for this useful catalogue.

### THE BEST EAU DE COLOGNE.

THE 4711 Eau de Cologne—of which I have received a sample—is so well known that it is impossible to say anything new in its praise. Those who purchase this delightful perfume are safe from disappointment, and it must always be welcome in the sick-room, where inferior perfumes are especially objectionable. The London depôt is at 62 New Bond Street. It can be obtained at all good chemists and elsewhere where toilet requisites are sold.

### Wants and Workers.

MISS E. L. C. EDEN, The Grange, Kingston, Taunton, would be glad to receive offers of copies of THE HOSPITAL and other books and magazines for the lending library for the use of Somersetshire district nurses.

### Echoes from the Outside World.

### Movements of Royalty.

On Saturday the King paid a visit to Compton Place, Eastbourne, and was received at Eastbourne Station by his host the Duke of Devonshire. His Majesty, with the Duke and Duchess, walked to St. Peter's Church on Sunday morning and attended service. Afterwards, in company with the Duchess, he drove along the parade. In the afternoon the house party drove in motor-cars to Beachy Head. The King returned to town on Monday morning, and in the afternoon he and the Queen travelled to Slough for the purpose of paying a visit to Eton College. Prior to the arrival of their Majesties at the College, the Prince and Princess, accompanied by the Princes Edward and Albert of Wales, were received with much enthusiasm by a large company of invited guests and the Eton boys, which was renewed when the coming of the King and Queen was heralded by the appearance of the scarlet outriders in the school yard. After the National Anthem had been sung, several presentations were made, and then followed the presentation of the addresses, which were bound in Eton blue silk. The latter were simply handed to the King, who smiled and bowed; and after that, at the bidding of Dr. Warre, the head master, there were rolling volleys of cheers from the boys. The chapel, schools, and, in fact, all the buildings having been inspected by the Royal visitors, who included Prince John of Glucksburg, the "procession of boats" took place, the series of aquatic evolutions being watched with much interest by the illustrious company in the pavilion. Finally, the Royal party embarked in the State barge for the Albert Bridge, whence they drove to Windsor Castle.

### The War in the Far East.

A Japanese force occupied Saimatse on Tuesday, last week, the Japanese losing in the encounter three men, besides 24 wounded. The Russians left 23 dead on the field. On the following day, General Kuroki, co-operating with the forces that landed at Ta-ku-shan, took possession of Sin-yen, and drove out the Russians with some loss. General Oku reports that the bodies of 10 Russian officers and 664 men found in the vicinity of Naushan have been carefully buried by the Japanese Military Administrative Commission and gendarmes. According to a Reuter despatch from Niu-chwang, the Japanese in the vicinity of Pu-lau-tien lured a Russian force on Saturday into making an attack, then took them in flank, and defeated them. The Russians are said to have lost 800 men. The Czar has received a telegram from General Stackelberg reporting that a battle was fought on Tuesday near Wa-fang-Kau, the Japanese attack being repelled, but a Russian general was wounded and a colonel killed.

### International Congress of Women.

A reception in the Philharmonic Concert Hall at Berlin on Sunday evening marked the opening of the International Congress of Women. It is estimated that the number of delegates is about 6,000, and they claim to represent some 7,000,000 women. Of these, 3,000 are foreigners, and the honorary president, Mrs. Susan B. Anthony, a lady of 84 years of age, is a native of the United States. The English delegates include Lady Aberdeen, Lady Marjorie Gordon, and Lady Battersea. At the meeting on Monday, Lady Aberdeen expressed her cordial recognition of the co-operation of German women in the Congress, and the gratitude of the assembly for the lively interest which the Empress displays in the objects of the movement. Speaking on "Woman as a Social Educator," Lady Aberdeen said that mothers must learn to increase their influence over their sons and daughters, and thus spread harmony abroad. Miss Teresa Wilson, an English delegate, gave an address on "Women as Farmers," and warmly recommended agricultural pursuits to strong women. On Tuesday morning the Empress received the leading representatives of the Congress, and expressed her satisfaction with its proceedings and with the way in which the foreign delegates had been received in Berlin. The proceedings of the day were largely occupied with the question of elementary education and with the lot of women employed in factories and workshops.

### Lady Curzon's Father.

The late Mr. Levi Leiter, who died last week rather suddenly from heart failure, was the father of Lady Curzon of Kedleston. Born in 1834, Mr. Leiter commenced life as an office boy in his native town, Leitersburg, Washington county. Subsequently, he was employed as clerk in various stores, and in the fifties he became a partner in a dry-goods firm at Chicago. Ultimately, he was able to obtain a controlling share in this business, and in the course of a few years amassed a fortune of many millions of dollars. In 1881 he retired from the business, and devoted his attention to speculations in real estate in Chicago, and to the management of the various companies with which he was connected. He leaves behind him a widow, one son, and three daughters.

### Arrival and Departure of "Dr." Dowie.

A person named John Alexander Dowie, who styles himself "Elijah the Restorer and General Overseer of the Christian Catholic Church in Zion" arrived in London on Saturday morning. As soon as he had exchanged greetings with his relatives he was handed a letter from the manager of one of the London hotels, stating that as "Dr." Dowie had been guilty of impolite references to the King it would be impossible for him to stay at that establishment. Finally the "General Overseer" from Zion City, Illinois, was obliged to repair to the suburban home of one of his elders for shelter. On Sunday "Dr." Dowie appeared at the headquarters of the Zionist movement in Euston Road, where admittance was by ticket only. The ticket holders, many of whom were cripples and some blind, were received by a dozen officials, five of whom were in the uniform of the Zion Guards, which resembles that of a prison warder, except that instead of a sword a bible is carried on the left hip. "Dr." Dowie gave addresses to his followers, and these were to have been continued on Monday, but the manager of the hotel discovering the personality of his guest, at once requested him to depart. As no other hotel would receive him, the "Doctor" cancelled all his engagements and left by the continental train.

### Crystal Palace Jubilee.

The jubilee of the Crystal Palace was celebrated on Saturday by a special musical festival. Sir August Manns, now nearly eighty, was the conductor. The programme commenced with Mendelssohn's "Hymn of Praise," and contained numbers by Wagner, Handel, Elgar, Sullivan, and Saint-Saëns. Mme. Albani sang the soprano music from the "Hymn of Praise," and the waltz from "Roméo et Juliette"; Miss Agnes Nicholls, besides taking the second part in the duet, "I waited for the Lord," gave the solo in "The night is calm," from "The Golden Legend"; Miss Muriel Foster was noteworthy in "Ché faro," and in the prayer from "The Golden Legend"; Mr. Ben Davies, as usual, was at his best in the "Watchman" scene of Mendelssohn's work; and Mr. Santley sang the "Evening Star" from Taunhaüser with his accustomed vigour. The singers and instrumentalists numbered something like 3,000 performers.

### A Book and its Story.

### THE WOMEN OF JAPAN.*

In a recent review of Mr. Diósy's book on Japan, "The New Far East" (June 4th), we mentioned that he acknowledged his indebtedness to Miss Bacon, an American writer, for much of the information in it which had reference to the position and training of Japanese girls and women. Having accidentally met with the book ourselves since reading "The New Far East" we feel that a further notice of these gentle and heroic little women, whose lives are so dissimilar to those of their sex in the Western hemisphere, may be interesting to Hospital readers. So little is known of them, and that little so misunderstood, that Miss Bacon was anxious that Japanese social life should be better comprehended through a further knowledge of the women of the country, among whom she has apparently lived, and of the homes which they make. One reason why the Japanese at home are so little known to foreigners is explained by the reticence which they preserve towards all that actually concerns them personally. "The Japanese under an appearance of frankness and candour hides an impenetrable reserve. . . . Only life in the home itself can show what a Japanese home may be; and only by intimate association—such as no foreign man can ever hope to gain—with the Japanese ladies themselves, can much be learned of the thoughts and daily lives of the best Japanese women. I have been peculiarly fortunate in having enjoyed the privilege of long and intimate friendship with a number of Japanese ladies who have spoken to me as freely, and shown the details of their lives as openly, as if bound by the closest ties of kindred. . . ."

The Japanese child comes into the world with perfect manners, which may be the product of untold centuries of careful training in the gentle art of self-effacement and consideration for others, or it may point to a more perfect civilisation than our own. "How much of the politeness of the Japanese is the result of training, and how much is inherited from generations of civilised ancestors, it is difficult to tell: babies are born into the world with a good start in gentle manners and the uniformly gentle and courteous treatment that they receive from those about them, together with the continual verbal teaching of the principle of self-restraint and thoughtfulness for others, produce with very little difficulty the universally attractive manners of the people." This charm of manner is not confined to public occasions, but is carried out in the daily relations of home life, and perhaps the following extract points to a condition of things which would particularly strike an American, from the very absence of it in a country where the rising generation have it all their own way. "One curious thing in a Japanese household is to see the formalities that pass between brothers and sisters, and the respect paid to age by every member of the family. The grandfather and grandmother come first of all in everything—no one at the table must be helped before them in any case; after them come the father and mother, and, lastly, the children, according to their ages. A younger sister must always pay respect to an elder, even in the matter of walking into the room before her. The wishes and convenience of the elder, rather than the younger, are to be consulted in everything, and this lesson must be learned early by children. As the Japanese child passes from girlhood to womanhood she has much affection and solicitous care bestowed upon her. She is trained in all the domestic and social duties which will fit her to take her place as the wife and mother of the household. She is instructed in books and mathematics form part of the educational code of the new woman. In all but the highest families the daughters of the house wait upon their parents' guests. If a guest is expected to whom particular honour is to be paid he dines separately with the father, and the daughter or wife wait on them. This is to show honour to him, and not for lack of servants, for there may be a staff within call ready to take the dishes from the hands of their mistress or her daughter. Then, again, in all but the most aristocratic families the housework of the simple Japanese dwelling is done by the daughters of the house. This is less arduous than in Western households, naturally, for without furniture, carpets, fireplaces or windows, etc., there is little to keep in order. Fashions do not change in Japan, so that women have one cause less for competition, and cooking is to a great extent done outside the house.

"The Japanese mother's life is one of perfect devotion to her children; she is their willing slave. Her days are spent in caring for them, her evenings in watching over them. In sickness and in health, day and night, the little ones are her one thought, and from the home of the noble to the humble cot of the peasant this tender mother-love may be seen in all its phases. Even with plenty of servants the mother performs nearly all the duties often delegated to nurses in this country. The father has little to do with the training of children, which is left entirely to the mother, and, except for the interference of the mother-in-law—a very formidable figure in domestic circles of which she is an invariable member—she has her own way in their training until they are long past childhood."

The Japanese now realise the impossibility of nursing cases of serious illness at home and gladly avail themselves of the hospitals and foreign system of medicine and nursing since their introduction into the country.

Eastern and occidental standards of female beauty are so opposite that it is amusing to read that the very babies will scream with horror at the first sight of a blue-eyed light-haired foreigner, and it is only after considerable familiarity with such persons that they can be induced to show anything but the wildest fright in their presence.

"To the Japanese, the ideal female face must be long and narrow; the forehead high—narrow in the middle, but widening—lowering at the sides, conforming to the outline of the beloved Fuji, the mountains that Japanese art loves to picture. Contrast this with blue eyes, set into deep sockets, the bridge of the nose rising as a barrier between them, which to the untravelled Japanese gives a fierce grotesqueness to the face and is singularly unattractive to them."

On the important subject of the education of Japanese girls on Western lines Miss Bacon has more to say than this brief notice of her work can include, but she sums up the question thus: "What are the effects produced so far by educating the Japanese woman to a point above the old Japanese standard? In many happy homes to-day we find husbands educated abroad, and knowing something of the home life of foreign lands, who have sought out wives of broad intellectual culture, and who make them friends and confidantes, not simply housekeepers and head-servants. In such homes the wife has freedom, not such as is enjoyed by American women perhaps, but equal to that of most European women. In such homes love and equality rule, and the power of the mother-in-law grows weak. These homes are sending out healthy influences that are daily having their effect, and raising the position of women in Japan."

* "Japanese Girls and Women." By Alice Mabel Bacon. (Gay and Bird. 5s.)

### Notes and Queries.

**FOR REGULATIONS SEE PAGE 143.**

*Home.*

(93) Can you tell me where an invalid lady, with weak heart, could be received at 10s. a week, near London preferred?—*V. A. B.*

Either the Woodside Home. Whetstone, N., or the Ladies' Home, 53 Abbey Road, St. John's Wood, might be suitable. Write for admittance to the latter to the Hon. Secretary, 75 St. George's Road, S.W. The charges here are somewhat more than stated, but they include medical attendance and medicines.

*Hospital Training.*

(94) A friend of mine, aged 20, wishes to get into a good hospital for three years' training. Please name some hospitals. Does the Brighton hospital take probationers, and is the training good?—*M. B. H.*

Your friend is too young excepting for a few of the children's hospitals. The Sussex County Hospital, Brighton, is a large training school, and receives probationers for a three years' course. Premium £35. See "The Nursing Profession: How and Where to Train," for list of training schools and full particulars.

I am a general servant and wish to become a nurse. I have been advised to go as wardmaid. Will you kindly tell me if it is usual for matrons to engage wardmaids as probationers?—*Enquirer* (Penrith).

Do not go as a wardmaid if you wish to become a nurse. Write to the matron of the largest infirmary near you, giving her full particulars, and enclosing stamp for reply. See reply to *M. B. H.* No premium is required by the Cumberland Infirmary, Carlisle, and the Royal Albert Edward Infirmary, Wigan. The salary at both begins at once.

Will you kindly tell me if Middlesbrough Infirmary is a recognised training school for nurses?—*Daisy.*

The North Riding Infirmary grants a three years' certificate. As the number of beds is less than 100, it would be as well, before entering, to write to the Local Government Board, Whitehall, S.W., and ask if the certificate is sufficient qualification for the post of superintendent nurse.

*Co-operative Societies.*

(95) Will you kindly tell me where I could find a list of co-operative nursing institutions?—*E. E.*

"The Nursing Profession: How and Where to Train" contains the names of these institutions.

*Registered.*

(96) I had nine months' training at the Nurses' Home, Plaistow, where I obtained the L.O.S. certificate, and have just completed my three years as a district nurse. Will you kindly tell me if it is necessary for me to be registered, and, if so, how must I proceed?—*E. J. H.*

You must be registered. Write to the Central Midwives Board, 6 Suffolk Street, Pall Mall.

Will you kindly tell me the cheapest and quickest way by which a midwife who has had many years' experience could obtain a certificate of competence?—*M. D.*

See reply to *E. J. H.* She is probably already qualified for registration as having been in *bona-fide* practice before the passing of the Act.

Can you give me any information regarding country midwives and where they may be registered? I have been trained in widwifery under a medical man, and have been in practice 18 months.—*D. O.*

See reply to *M. D.* and *E. J. H.*

**Important Nursing Textbooks.**

"Nursing Profession: How and Where to Train." 2s. net; 2s. 4d. post free.

"Nurses' Dictionary of Medical Terms and Nursing Treatment." By Honnor Morten. 2s. post free.

"Nursing." By Isabel Hampton. 7s. 6d. net; 7s. 10d. post free.

"Surgical Ward-Work and Nursing." By Dr. Alex. Miles. 3s. 6d. net; 3s. 10d. post free.

"Outlines of Insanity." By F. H. Walmsley, M.D. Popular Edition. 1s. 6d. post free.

"Notes on Pharmacy and Dispensing for Nurses." By C. J. S. Thomson. 1s. post free.

The above works are obtainable of all booksellers, or direct from The Scientific Press, Limited, 28 & 29 Southampton Street, Strand, London, W.C.

### For Reading to the Sick.

"PEACE, BE STILL."

AND all the ministers of peace were there;
Faith, Hope, and Love, and all the starry host
Of Angels, and the rulers of the calm.
And all looked down, leaning o'er heavenly walls—
The battlements of Heaven itself—to watch
One tiny skiff that tossed upon the flood
Of the great world-sea, while the mighty waves
Were gath'ring all their strength.
For still the bark
Rose on each billow. Though the thund'rous shock
Of warring waters filled the air with foam,
Still she was safe. . . .
That Hand was outstretched now, as once before,
When ruling the tumultuous water-floods
In Galilee; and well the Angels knew,
And all the powers in Heaven, and all its host,
That neither force of wind or wave, nor strength
Of adverse spirit, could prevail to drown
The little bark o'er which that Hand was held.

*T. V. Fosbery.*

If the vessel of our soul be tossed with winds and storms, let us awake the Lord, Who reposes in it, and He will quickly calm the sea.—*Brother Lawrence.*

The Lake of Galilee, near which our Lord spent so much of His time, is exposed to great storms, which are the more terrible because they rise so suddenly and unexpectedly. Picture the Apostles in the boat with their Master; He is "in the hinder part of the ship, asleep upon a pillow."

"The disciples awaked Him," saying, "Lord, save us, we perish." They ought to have known that with our Lord they were safe; they ought to have known that He knew all about the storm, and would be equal to the occasion, for it is His will "that not one of His little ones should perish." And this is precisely what our Lord told them: "Why are you fearful, O ye of little faith," He said, as He awoke from sleep. "And rising up, He commanded the winds and the sea, and there came a great calm."

"O ye of little faith;" the lesson lies in that epithet "little." Our lives are as a sheet of water exposed to the winds and rains of many a trouble and temptation. Sometimes we are warned of their approach, but often we have no warning, yet are bidden be ready. Can we believe and act as though we knew that our Lord was standing by us, taking note of everything, that He has us really by the hand to help us?

If He is there, why does He not heal me? If He loves me, why does He not calm the storm? If He values me, why does He not make my way through the waters of life more smooth and plain? Why is He "asleep?" Does Our Lord then "turn and look on us," as on St. Peter, and say "Why are you fearful, O ye of little faith?" We do trust Our Lord; we do believe in Him; but is our faith great and fire-tried, or "little" and unequal to the storm?

"The winds and the sea obey Him." He is the Lord, and the day of calm comes according to His good will. Let us trust Him that never beyond what we are able to bear shall the winds and waves of trial beat upon us. So we end at the feet of our Lord, asking His help in all difficulties and assuring Him that we trust Him never to allow us to suffer shipwreck, "being confident of this very thing that He who hath begun a good in us will perfect it unto the day of Christ Jesus."—*Anon.*

## Figures and Tables

**Figure f1:**